# Left ventricular fluid kinetic energy time curves in heart failure from cardiovascular magnetic resonance 4D flow data

**DOI:** 10.1186/s12968-015-0211-4

**Published:** 2015-12-20

**Authors:** Mikael Kanski, Per M. Arvidsson, Johannes Töger, Rasmus Borgquist, Einar Heiberg, Marcus Carlsson, Håkan Arheden

**Affiliations:** Department of Clinical Physiology, Clinical Sciences, Lund University, Lund University Hospital, Lund, Sweden; Department of Cardiology, Clinical Sciences, Lund University, Lund University Hospital, Lund, Sweden; Department of Biomedical Engineering, Faculty of Engineering, Lund University, Lund, Sweden; Centre for Mathematical Sciences, Faculty of Engineering, Lund University, Lund, Sweden

**Keywords:** Cardiovascular magnetic resonance, 4D-flow, Heart failure, Kinetic energy, Diagnosis

## Abstract

**Background:**

Measurement of intracardiac kinetic energy (KE) provides new insights into cardiac hemodynamics and may improve assessment and understanding of heart failure. We therefore aimed to investigate left ventricular (LV) KE time curves in patients with heart failure and in controls.

**Methods:**

Patients with heart failure (*n* = 29, NYHA class I-IV) and controls (*n* = 12) underwent cardiovascular magnetic resonance (CMR) including 4D flow. The vortex-ring boundary was computed using Lagrangian coherent structures. The LV endocardium and vortex-ring were manually delineated and KE was calculated as ½mv^2^ of the blood within the whole LV and the vortex ring, respectively.

**Results:**

The systolic average KE was higher in patients compared to controls (2.2 ± 1.4 mJ vs 1.6 ± 0.6 mJ, *p* = 0.048), but lower when indexing to EDV (6.3 ± 2.2 μJ/ml vs 8.0 ± 2.1 μJ/ml, *p* = 0.025). No difference was seen in diastolic average KE (3.2 ± 2.3 mJ vs 2.0 ± 0.8 mJ, *p* = 0.13) even when indexing to EDV (9.0 ± 4.4 μJ/ml vs 10.2 ± 3.3 μJ/ml, *p* = 0.41). In patients, a smaller fraction of diastolic average KE was observed inside the vortex ring compared to controls (72 ± 6 % vs 54 ± 9 %, *p* < 0.0001). Three distinctive KE time curves were seen in patients which were markedly different from findings in controls, and with a moderate agreement between KE time curve patterns and degree of diastolic dysfunction (Cohen’s kappa = 0.49), but unrelated to NYHA classification (*p* = 0.12), or 6-minute walk test (*p* = 0.72).

**Conclusion:**

Patients with heart failure exhibit higher systolic average KE compared to controls, suggesting altered intracardiac blood flow. The different KE time curves seen in patients may represent a conceptually new approach for heart failure classification.

## Background

Heart failure (HF) is a common disease [1] with high mortality [2]. Accurate diagnosis of HF remains a challenge, and available diagnostic tests are classified as “Level C” in the European Society of Cardiology Guidelines for Heart Failure [3], i.e. current methods are based on expert consensus, small and/or retrospective studies, and registry analysis. Therefore, new quantitative measures of HF are needed.

Blood flow patterns in the left ventricle (LV) are closely connected to the shape and motion of the myocardium, valves and great vessels, and may therefore be a sensitive marker of cardiac function and dysfunction, including HF [4]. Kinetic energy (KE) is a quantitative measure of LV blood flow which is connected to diastolic function [5, 6] and is altered in HF [4, 7]. Previous studies have shown that KE increases in mild to moderate heart failure compared to healthy controls [4]. However, KE has not been studied in more advanced stages of HF.

Furthermore, one of the most prominent features of LV blood flow is the formation of a vortex ring downstream from the mitral valve during early rapid filling [8, 9]. Recent studies have shown that features of the vortex ring are connected to diastolic function and dysfunction [10]. Diastolic dysfunction is a condition with poor prognosis even in its mild form [11], which contributes to worsening cardiac performance in the downward spiral of HF. Investigating the connection between vortex ring formation, diastolic dysfunction and KE may therefore lead to new better understanding of HF.

Therefore, the aim of this study was to quantify LV KE in patients with moderate and severe heart failure. Furthermore, we aimed to investigate the relationship between KE and diastolic vortex ring formation, as well as cardiac dyssynchrony and current clinical measures of HF.

## Methods

### Study design and population characteristics

The regional ethics committee in Lund, Sweden, approved the study and all subjects provided written informed consent. We prospectively included 29 patients with NYHA class I-IV heart failure, and 12 healthy controls. Patient inclusion criteria were LV ejection fraction (EF) < 40 % and optimal heart failure medication. Exclusion criteria were severe renal failure (GFR < 30 ml/min), unstable angina pectoris or acute myocardial infarction within 30 days prior to inclusion, chronic atrial fibrillation, significant valvular disease, or pregnancy. Ischemic etiology to heart failure was defined prior to cardiovascular magnetic resonance (CMR), by history of previous angina and detection of cardiac-specific Troponin, and previous percutaneous coronary intervention (PCI) or coronary artery bypass-graft (CABG). No history of previous angina, PCI, or CABG resulted in the diagnosis dilated cardiomyopathy (DCM). Controls were free from medication and had no history of cardiovascular or systemic disease. Population characteristics are shown in Table 1, and patient characteristics are shown in Table 2. Ten out of 12 controls were part of a previous validation study [5]. All subjects underwent CMR at 1.5 or 3 T using Philips Achieva systems (Philips Medical Systems, Best, the Netherlands). Patients also underwent a standardized 6-minute walk test (6MWT).

**Table 1:** Study population characteristics

	Heart failure	Controls	*p*-value
Male sex (*n*, [%])	24 [83 %]	8 [67 %]	0.41 ^a^
Age (years)	67 ± 8	27 ± 3	<0.0001
BSA (m^2^)	2.0 ± 0.2	1.9 ± 0.3	0.63
Heart rate (bpm)	68 ± 15	62 ± 7	0.21
LV EDV (ml)	348 ± 110	197 ± 45	<0.0001
LV ESV (ml)	264 ± 109	76 ± 22	<0.0001
LV SV (ml)	84 ± 23	121 ± 27	0.0001
LV EF (%)	26 ± 8	62 ± 5	<0.0001
Cardiac index (ml/min/m^2^)	2.6 ± 0.6	3.7 ± 0.7	<0.0001

*BSA* body surface area (Mosteller); *bpm* beats per minute; *LV* left ventricular; *EDV* end-diastolic volume; *ESV* end-systolic volume; *SV* stroke volume; *EF* ejection fraction

^a^ Calculated using Fisher’s exact test, all other using Mann–Whitney *U*-test

**Table 2:** Patient characteristics and medication

Etiology (*n*, [%])	
- IHD	18/29 [62 %]
- DCM	11/29 [38 %]
LBBB (*n*, [%])	23 [79 %]
Diabetes (*n*, [%])	4 [14 %]
Medication (*n*, [%])	
- Betablocker	27 [93 %]
- ACEI or ARBs	29 [100 %]
- Trombocyte-aggregation inhibitors	18 [62 %]
- Diuretics	20 [69 %]
- Statins	19 [66 %]

*IHD* ischemic heart disease; *DCM* dilated cardiomyopathy; *ACEI* angiotensin-converting enzyme inhibitors; *ARBs* angiotensin II receptor blockers

### CMR parameters

Eight out of 12 controls (67 %) and 21/29 patients (72 %) underwent CMR at 1.5 T. A 32-channel cardiac coil was used at 1.5 T and a 6-channel cardiac coil was used on 3 T. For all subjects, balanced steady-state free precession (bSSFP) cine images were acquired in standardized short-axis and long-axis views, and three-dimensional, time-resolved phase contrast images (4D flow) were acquired covering the entire heart. Patients underwent a clinical CMR exam for viability assessment and determination of regional and global LV function.

*4D flow*: A segmented gradient echo sequence, 2 views per segment, with retrospective ECG triggering and a SENSE parallel imaging factor of 2 was used as previously described [5]. 4D flow was acquired during free breathing with a field of view covering the entire heart, without respiratory gating, as previously validated [12, 13]. The acquired temporal resolution was typically 50–55 ms, rendering 10–21 phases depending on heart rate, and was reconstructed to 40 heart phases. Typical scanning parameters were TE/TR: 3.7/6.2 ms, flip angle: 8°, acquired and reconstructed voxel size: 3x3x3 mm, VENC 100 cm/s. Concomitant gradient terms were compensated by the CMR scanner. Phase unwrapping was performed in two stages: 1) an automated algorithm was performed on the entire 4D dataset using a plugin for Segment 1.9 (Medviso, Lund, Sweden) [14]; 2) the dataset was visually inspected for residual phase wraps, and these were corrected in all datasets when deemed corrigible. Phase background errors (e.g. due to eddy currents) were corrected offline by subtraction of a linear fit of velocities in stationary tissue, as previously validated [5, 12, 13].

**Fig. 1:**
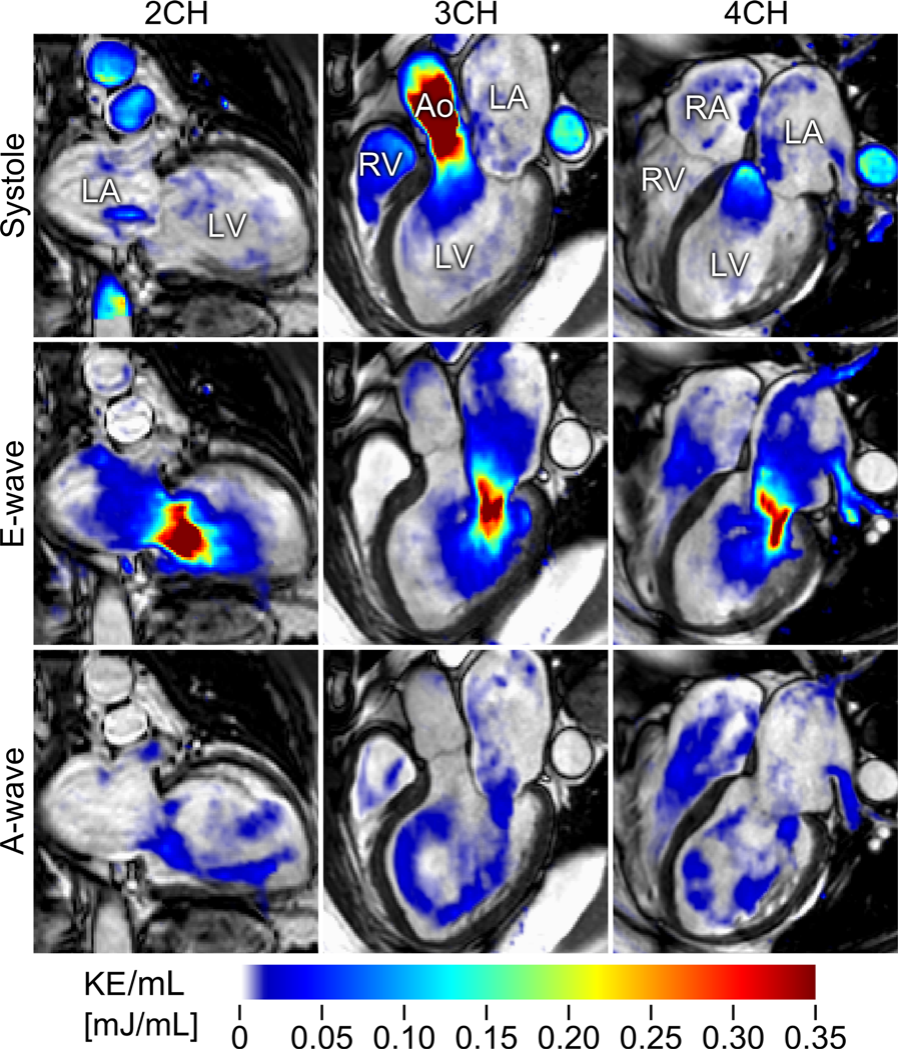
An example of kinetic energy (KE) in a patient with heart failure at three time-points during the cardiac cycle: peak systole (*top panel*), peak early diastolic filling (*middle panel*), and during atrial systole (*bottom panel*). Left column shows 2-chamber view (2CH), middle column shows 3-chamber view (3CH), and right column shows 4-chamber view (4CH). LA: Left atrium; LV: Left ventricle; RA: Right atrium; RV: Right ventricle; Ao: Aorta

*LV volumetrics and KE analysis*: The LV was defined by manually drawing the contours of the blood volume in short-axis slices over the entire cardiac cycle in cine images acquired with retrospective ECG triggering (typical scanning parameters: TR/TE: 1.4/2.8, flip angle 60°, in-plane spatial resolution 1.3x1.3 mm, temporal resolution 30 ms, slice thickness: 8 mm, no slice gap). Left ventricular mass (LVM), end-diastolic volume (EDV), and end-systolic volume (ESV) were measured by manual delineation of endocardial and epicardial borders of the LV. The papillary muscles were excluded in LVM measurements. Stroke volume (SV) was defined as EDV-ESV. The peak emptying rate (PER, ml/s) and peak filling rate (PFR, ml/s) were acquired from the time-resolved short-axis delineations of the LV. The delineations were transferred to the 4D data set and KE was calculated as the sum of ½mv^2^ for each voxel [15], where m is the mass of blood in one voxel (density of blood is assumed to be 1050 kg/m^3^ [16]) and v is the velocity magnitude in each voxel. The KE for each time step was computed as the sum of KE in all voxels inside the LV endocardial delineations. The plotted KE time curve patterns were visually assessed for each patient and grouped by appearance by two observers and arrived at a consensus description of the characteristics of each group. Thereafter, a third observer grouped the KE time curves according to these predefined criteria. This resulted in the same grouping as suggested by observers 1 and 2. Figure 1 shows KE visualization in one patient with heart failure. Temporal average KE was computed separately for systole and diastole.

*Kinetic energy terminology was defined as follows*: “KE time curve” = the amount of KE inside the LV for each time step over the entire cardiac cycle; “Systolic average KE” = average of KE for all systolic time steps; “Diastolic average KE” = average of KE for all diastolic time steps.

**Fig. 2:**
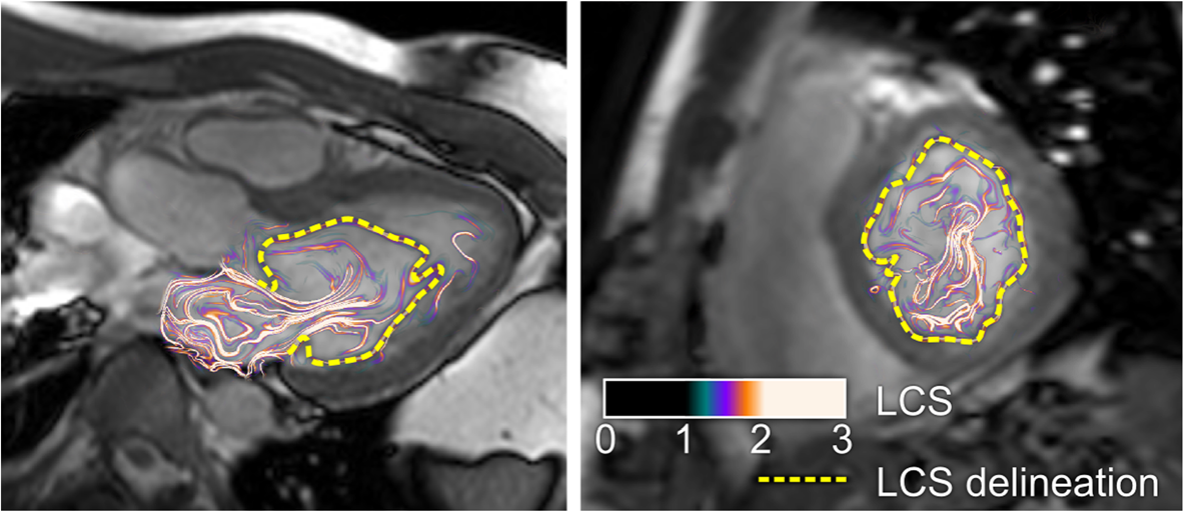
Vortex-ring delineation using Lagrangian coherent structures (LCS). Left: LCS in 3-chamber view of the LV. Right: LCS in short-axis view. Delineation of LCS for each diastolic time frame during enables quantification of blood inside and outside the diastolic vortex

*Vortex ring size and vortex KE*: In order to delineate the vortex ring formed from blood flowing into the LV during diastole [8, 9], Lagrangian coherent structures (LCS, Fig. 2) were computed from 4D flow data as previously described [8] and validated [13]. Particle-tracing computations required for the LCS algorithm were implemented in CUDA-C and performed on Graphical Processing Unit (GPU) cards (NVIDIA, Santa Clara, CA, USA). The limits of the vortex ring were determined as follows: LCS indicative of vortex-ring formation were delineated in short-axis images for each time step in diastole. The vortex ring delineations were used to differentiate the vortex volume for each time step and KE inside and outside of the vortex ring for each time step. During the entire diastole, the change in KE (ΔKE) inside the diastolic vortex was calculated as the maximum change in KE from the formation of the diastolic vortex to end-diastole. Also, ΔKE inside the vortex was calculated during diastasis (between E-wave and A-wave).

*Terminology for vortex KE was defined as follows*: “KE inside vortex” = average of KE in all voxels inside the diastolic vortex; “KE outside vortex” = average of KE in all voxels outside the diastolic vortex.

*Viability analysis*: ECG-triggered late gadolinium enhancement (LGE) imaging was performed with a 3D inversion recovery (IR) gradient echo (GRE) sequence in mid-diastole. Images were acquired in short-axis slices covering the entire LV, and in long-axis views. LGE imaging was performed 10–20 min after intravenous administration of 0.2 mmol/kg gadolinium-based CMR contrast agent (Dotarem, Guerbet, Roissy, France). Typical scanning parameters were: TE 1.3 ms, effective repetition time every heartbeat, 5 slices per breath-hold, flip angle 15°, slice thickness 8 mm, no slice gap, and in-plane resolution 1.5x1.5 mm. Inversion time was chosen to optimally null the myocardium. LGE was quantified using a semi-automatic weighted algorithm with manual corrections as previously described [17].

*Diastolic function*: Diastolic function in patients was assessed using the following measures by CMR: early and atrial mitral inflow ratios (E/A), mitral E-wave deceleration time (MDT), E-wave peak velocity, velocity of the LV myocardium in early diastole (mean E’), E/E’, maximum left atrial volume normalized to BSA, and pulmonary venous flow profile [18]. E, A, and pulmonary vein flow profiles were extracted from the 4D flow data, and E’ was measured in the CMR 4-chamber view. Maximum left atrial volume was measured by manual planimetry in short-axis cine images.

*Dyskinesia*: Dyskinesia was assessed in patients using CMR. Dyskinesia was visually assessed based on the presence of septal segments with dyskinesia, from 0 segments = no dyskinesia, to 1–4 septal segments = dyskinesia.

### Image analysis

All image analysis and visualization was performed using Segment 1.9 (Medviso, Lund, Sweden) [14]. Computations of LCS and KE were performed using in-house designed plugins for Segment [8, 15].

### Statistical analysis

Continuous variables are presented as mean ± SD unless otherwise stated. The following statistical analyses were performed using GraphPad Prism 6.04, (GraphPad Prism Software Inc, La Jolla, CA; USA): Comparisons between patients and controls using Mann–Whitney *U* test, Pearson’s correlation analysis, comparisons between KE time curve pattern groups using Kruskal-Wallis test, and agreement of rating of diastolic dysfunction using Cohen’s kappa. The variability in the KE time curve patterns was assessed using the coefficient of variation [19] calculated as SD/mean. IBM SPSS Statistics for Windows v21 (IBM, Armonk, NY; USA) was used for stepwise multiple regression analysis. The parameters for comparison with diastolic average KE were LVM, SV, PFR, and heart rate. For possible predictors of KE in patients with ischemic heart disease (IHD), EDV and fraction of ischemic scar relative to total amount of LV myocardium (%scar) were used. Differences with a *p*-value <0.05 were considered statistically significant. For entry into the regression models, *p* < 0.10 was used, and for removal *p* > 0.15.

## Results

Characteristics and LV volumes for patients and controls are presented in Table 1. Patients were older and had larger LV volumes and lower EF compared to controls. Further patient characteristics are listed in Table 2. IHD was the most common etiology (62 %) and patients used heart failure medications according to international guidelines [20].

### Temporal average kinetic energy

**Fig. 3:**
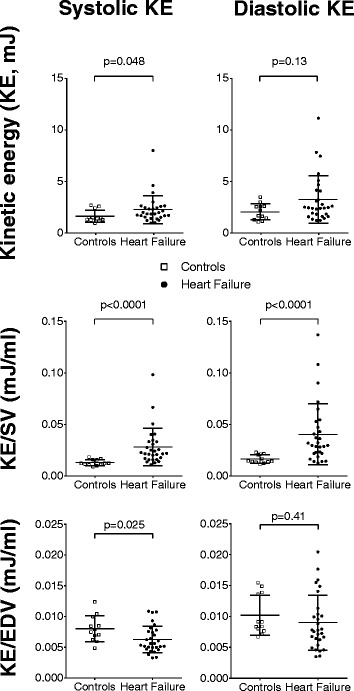
Systolic average kinetic energy (KE, *left column*), and diastolic average KE (*right column*) in controls (open boxes) and patients with heart failure (*black circles*). On the Y-axis, the upper panel of graphs shows KE; middle panel shows KE indexed to stroke volume (SV); and lower panel shows KE indexed to end-diastolic volume (EDV). Error bars show mean ± SD

There was no statistically significant difference in diastolic average KE between patients with heart failure and controls (3.2 ± 2.3 mJ vs 2.0 ± 0.8 mJ, *p* = 0.13). For systolic average KE, a statistically significant difference was seen (2.2 ± 1.4 mJ vs 1.6 ± 0.6 mJ, *p* = 0.048) (Fig. 3, *top panel*). Indexing KE to SV showed that patients had higher values compared to controls both for systolic average KE/SV (28.3 ± 18.4 μJ/ml vs 12.9 ± 2.9 μJ/ml, *p* < 0.0001) and diastolic average KE/SV (40.8 ± 29.7 μJ/ml vs 16.3 ± 4.1 μJ/ml, *p* < 0.0001) (Fig. 3, *middle panel*). When indexed to EDV, patients showed lower systolic average KE (6.7 ± 1.9 μJ/ml vs 8.0 ± 2.1 μJ/ml, *p* = 0.025), but no difference in diastolic average KE (9.0 ± 4.4 μJ/ml vs 10.2 ± 3.3 μJ/ml, *p* = 0.41) (Fig. 3, *bottom panel*).

### Determinants of diastolic average KE in patients

Multiple stepwise linear regression analyses showed LVM and peak filling rate (PFR) to be determinants of diastolic average KE (Table 3) and temporal average KE inside the diastolic vortex (Table 4), respectively. Diastolic average KE depended neither on heart rate nor on SV.

**Table 3:** Predictors of diastolic KE

Independent variables	Beta-value	*p*-value
LVM	0.50	0.003
PFR	0.31	0.051
Excluded variables	Partial correlation	
HR	0.30	0.13
LVSV	<0.01	1.00

Results from a stepwise multiple linear regression model for prediction of diastolic KE by independent variables in patients. LVM was the strongest predictor of diastolic KE, and peak filling rate (PFR) contributed to the model. Diastolic KE was not dependent on heart rate (HR) or stroke volume (LVSV)

**Table 4:** Predictors of KE inside diastolic vortex

Independent variables	Beta-value	*p*-value
LVM	0.43	0.008
PFR	0.42	0.01
Excluded variables	Partial correlation	
HR	0.30	0.12
LVSV	0.03	0.90

Results from stepwise multiple linear regression model for prediction of KE inside diastolic vortex by independent variables in patients. LVM was the strongest predictor of KE inside diastolic vortex, and peak filling rate (PFR) contributed to the model. KE inside diastolic vortex was not dependent on heart rate (HR) or stroke volume

### Correlation between KE and level of septal dyskinesia

Seventeen out of 29 patients had at least 2 dyskinetic septal segments (2 segments: 4 patients; 3 segments: 7 patients; and 4 segments: 6 patients). In patients with septal dyskinesia, there was no difference in diastolic average KE compared to patients without dyskinesia (Mann–Whitney *p* = 0.89). Furthermore, Kruskal-Wallis test showed no difference in diastolic average KE between patients with 0, 2, 3, or 4 dyskinetic septal segments (*p* = 0.76).

### Kinetic energy in patients with ischemic heart disease

Multiple stepwise linear regression identified the EDV as an independent predictor of temporal average KE in the LV (R^2^ = 0.28, *p* < 0.05). Percentage of scarred LV myocardium (%scar) did not contribute further to the model (Table 5).

**Table 5:** Predictors of temporal average KE in patients with ischemic heart disease

Independent variables	Beta-value	*p*-value
LV EDV	0.53	0.02
Excluded variables	Partial correlation	
Percent scarred LV	0.20	0.45

Stepwise multiple linear regression for prediction of temporal average KE by independent variables in *n* = 18 patients with heart failure due to ischemic heart disease. Temporal average KE depends on heart size (LV EDV), but not on the percentage of scar in the LV

### KE time curve analysis

**Fig. 4:**
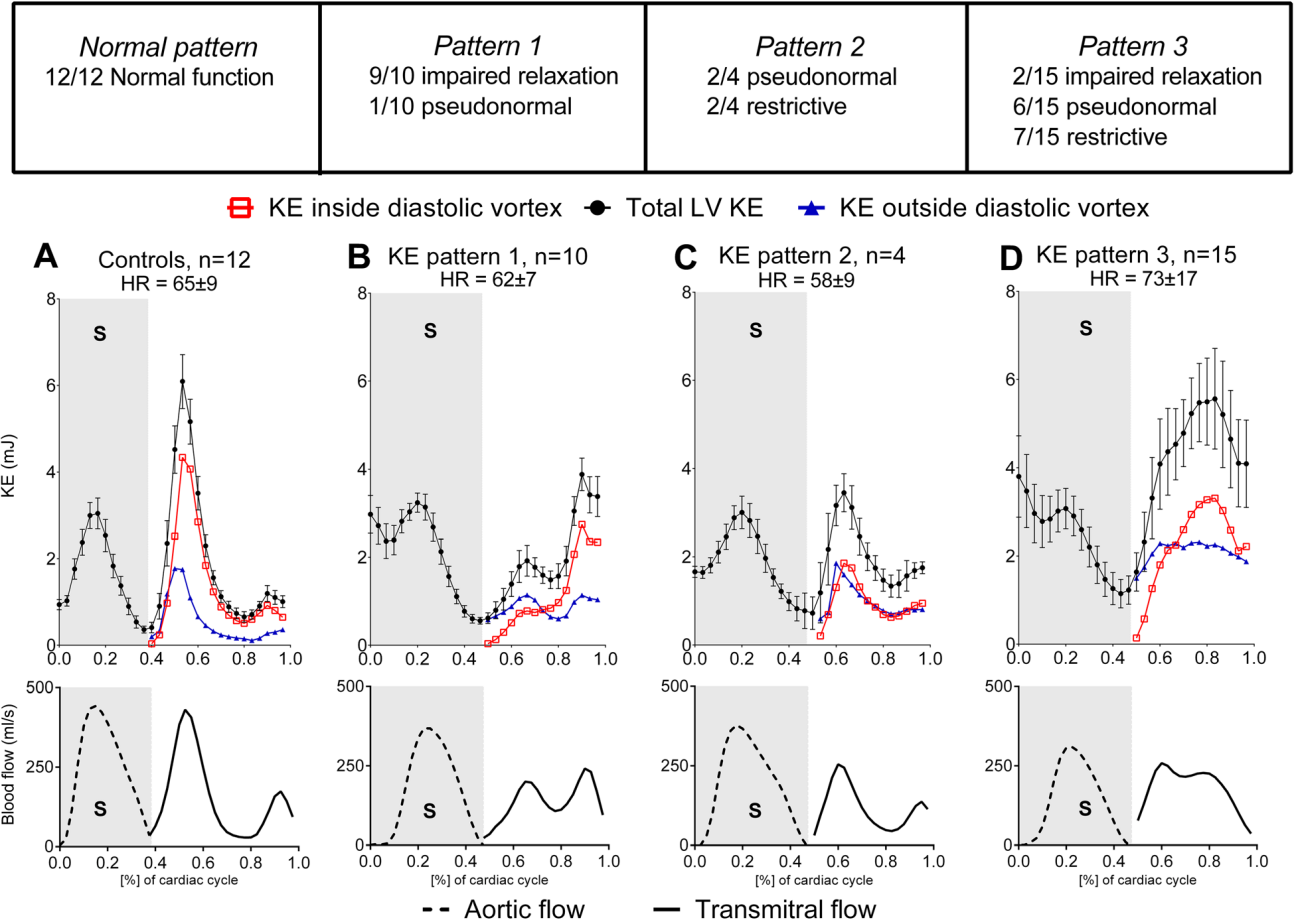
Top panel: Variation of intracardiac KE during the cardiac cycle, starting at end-diastole. KE ± SEM is shown for controls (**a**) and patients with three different time curve patterns (**b**-**d**). Black circles indicate KE (mJ) inside the entire left ventricle, open red boxes indicate KE (mJ) inside of diastolic vortex, and blue triangles KE (mJ) outside of the vortex. Bottom panel: Aortic flow curves, from end-diastole to end-systole, and transmitral flow curves, starting at end-systole. *KE* = kinetic energy; *HR* = heart rate; *LV* = left ventricle; *S* = systole. Error bars are omitted from KE inside and outside of the diastolic vortex as well as for flow curves for clarity

**Fig. 5:**
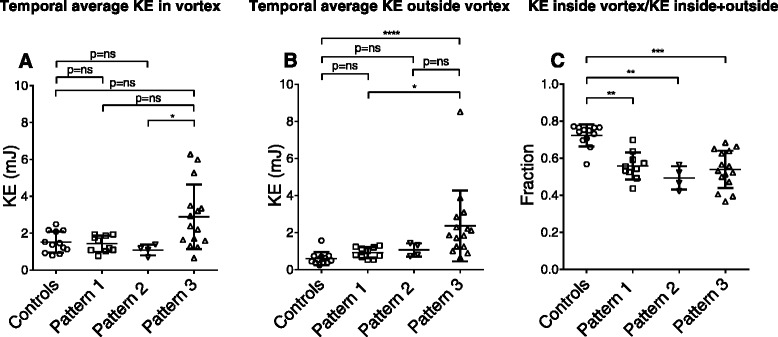
Temporal average kinetic energy (KE) inside the diastolic vortex (panel **a**) and outside the diastolic vortex (panel **b**) for controls and patients with heart failure subdivided by KE time curve patterns. Panel (**c**) shows the ratio between temporal average KE inside of the diastolic vortex and temporal average KE inside + outside of the diastolic vortex. This shows that patients with heart failure have lower fraction of KE inside of the diastolic vortex compared to controls. Comparisons are performed using Kruskal-Wallis test. Error bars show mean±SD. * = *p* < 0.05; ** = *p* < 0.01; *** = *p* < 0.001; ****p < 0.0001

Controls presented similar appearance of KE time curve patterns as previously described [15]; a peak during systole, a peak during the early rapid filling phase of diastole (E-wave), and a small, late peak separated from the first, corresponding to the atrial contraction (A-wave) (Fig. 4, panel a). There was no statistically significant difference in systolic or diastolic KE peak values between patients with heart failure and controls (Table 6). There was a moderate correlation between the change in diastolic KE and the change in transmitral flow (*R* = 0.55, *p* < 0.0001). The majority of total KE was found within the vortex ring in healthy controls (72 ± 6 %, Fig. 5). Patients had less KE inside the vortex ring compared to controls (time curve pattern 1: 56 ± 7 %, *p* < 0.01; time curve pattern 2: 49 ± 6 %, *p* < 0.01; time curve pattern 3: 54 ± 10 %, *p* < 0.001; Fig. 5), meaning that intracardiac diastolic average KE in heart failure to a higher extent resides outside the diastolic vortex. Throughout diastole, ΔKE inside the vortex correlated to Δvortex volume (R^2^ = 0.19, *p* = 0.018 for patients, R^2^ = 0.57, *p* = 0.0044 for controls). Analysis of diastasis only (excluding E-wave and A-wave) revealed similar correlations (R^2^ = 0.26, *p* = 0.0048 for patients vs R^2^ = 0.48, *p* = 0.0131 for controls). Since KE = ½mv^2^, this means that in patients, the change in KE inside the vortex can be explained to a higher degree by the change in velocity squared than by change in volume. However, in controls, the change in KE is about equally explained by change in volume and by change in velocity squared.

**Table 6:** KE values

	Heart failure	Controls	*p*-value
	(*n* = 29)	(*n* = 12)	
Systolic average KE (mJ)	2.2 ± 1.4	1.6 ± 0.6	0.048
Diastolic average KE (mJ)	3.2 ± 2.3	2.0 ± 0.8	0.13
Systolic average KE/SV (μJ/ml)	28.3 ± 18.4	12.9 ± 2.9	<0.0001
Diastolic average KE/SV (μJ/ml)	40.8 ± 29.7	16.3 ± 4.1	<0.0001
Systolic average KE/EDV (μJ/ml)	6.3 ± 2.2	8.0 ± 2.1	0.025
Diastolic average KE/EDV (μJ/ml)	9.0 ± 4.4	10.2 ± 3.3	0.41
Systolic KE peak (mJ)	3.3 ± 1.4	3.3 ± 1.2	0.92
Diastolic KE peak (mJ)	6.3 ± 4.0	6.4 ± 2.1	0.29

Values are presented as mean ± SD

### KE time curve appearances and comparison to diastolic function

Patterns in the appearance of the diastolic part of the KE time curves were identified. Since the early and late diastolic KE peaks depend mainly on velocity (½mv^2^) and coincide with the early diastolic filling (corresponding to E-wave) and atrial contraction (corresponding to A-wave), three main KE time curves in heart failure patients were discerned: 1) lower KE during early diastolic filling (corresponding to E-wave) compared to atrial contraction (corresponding to A-wave); 2) higher KE during the E-wave compared to during the A-wave; and 3) partial or complete fusion of the KE peaks during early diastolic filling and atrial contraction.

*Time curve pattern 1:* 10 out of 29 patients (34 %) (Fig. 4, panel b). Within time curve pattern 1, 7 patients (70 %) were characterized as having impaired relaxation, and 3 patients (30 %) as pseudonormal filling by CMR.

*Time curve pattern 2:* 4 out of 29 patients (14 %) (Fig. 4, panel c). Within time curve pattern 2, 2 patients (50 %) were characterized as having pseudonormal filling, and 2 patients (50 %) as restrictive diastolic dysfunction by CMR.

*Time curve pattern 3:* 15 out of 29 patients (52 %) (Fig. 4, panel d). A distinct spill-over of late diastolic KE is seen during the onset of systole. Within time curve pattern 3, 1 patient (7 %) was classified as impaired relaxation, 4 patients (27 %) as pseudonormal filling, and 6 patients (40 %) as restrictive diastolic dysfunction by CMR. In 4 patients (27 %) the degree of diastolic dysfunction could not be determined. Kruskal-Wallis test showed no statistically significant difference in heart rate between controls, time curve pattern 1, 2, or 3 (65 ± 9, 62 ± 7, 58 ± 9, 73 ± 17 bpm, *p* = 0.08). 11 out of 15 patients in pattern 3 were in the same heart rate range as pattern 1.

None of the patients were classified as having normal KE time curve pattern. There was a moderate correlation between KE time curve pattern and degree of diastolic dysfunction by CMR (Cohen’s kappa = 0.41, SE 0.14, 0.95 CI: 0.14–0.67).

The coefficient of variability of the KE time curve patterns was lower in patients compared to controls (0.60 ± 0.14 vs 0.89 ± 0.12, *p* < 0.0001) meaning that the KE time curves in patients were more flat compared to controls, who have distinct peaks in the KE time curves.

### KE time curves vs functional assessment

Median NYHA classification in time curve pattern 1, 2, and 3 were NYHA II, III, and III, respectively. Kruskal-Wallis test showed no statistically significant difference in NYHA classification between the three KE time curve patterns (*p* = 0.12). In time curve pattern 2, only one patient underwent 6-minute walk test (6MWT). Mann–Whitney *U* test showed no statistically significant difference in 6MWT between patients in time curve pattern 1 and time curve pattern 3 (443 ± 227 meters vs 420 ± 217 meters*, p* = 0.72).

## Discussion

This study has demonstrated that the LV systolic average KE is higher in patients with heart failure compared to controls, but lower when indexing to heart size. No difference was seen in diastolic average KE, however, a larger fraction of the diastolic KE was found outside the diastolic vortex in patients. Furthermore, patients with heart failure can be subdivided into three different KE time curve patterns, markedly altered compared to KE curve pattern in controls.

### Relation to earlier studies

In this study, patients with heart failure showed higher systolic and diastolic average KE/SV compared to controls, which is supported by findings by Eriksson et al*.* [4] who found a higher mean KE/ml in patients with mild to moderate heart failure. However, Eriksson et al. included patients with higher EF than in our study (42 ± 5 % vs 25 ± 8 %).

Our results indicate that patients with heart failure have altered intracardiac energy levels compared to controls. Moreover, this study presents three distinctive KE time curve patterns in heart failure, all markedly altered compared to the KE time curve pattern presented by controls as shown in this and previous work [7, 15].

When studying ventricular KE in healthy volunteers, Carlsson et al*.* found the LV diastolic KE higher compared to the right ventricle [15]. This was hypothesized to be explained by the “spring” mechanism during diastolic relaxation of the myocardial protein titin, which was later supported by KE analysis of the atrial blood pool [6]. This spring mechanism results in a drop in intraventricular LV pressure which in turn leads to suction of blood from the left atrium to the LV [6]. The LVM has been found to be an independent predictor of early diastolic peak KE [21]. This early diastolic suction force is reduced in diastolic dysfunction. Therefore, the observed difference in determinant of intracardiac KE between the healthy heart and the failing heart may partially be due to the remodeled dysfunctional LV myocardium. When this spring mechanism is lost, the failing heart will depend on compensatory mechanisms such as increased filling pressure in order to maintain an adequate cardiac output. We hypothesize that the shift from LV suction in the healthy heart to increased preload and changed diastolic inflow to the LV in the failing heart explains the altered KE time curves. However, the peak values in this study did not differ between patients and controls.

### Pathophysiological implications

The study of temporal average KE and KE time curves has potential to yield new insight and understanding of the pathophysiology in heart failure. In the human heart, altered energy levels may contribute to changes in pressure gradients that may lead to a slow remodeling of the myocardium that is seen in heart failure [22]. This effect is likely to be small at rest, and possibly more important during exercise, as previously suggested using simulations by Carlsson et al*.* [15]. As a consequence to the failing left ventricle, the pressure in the left atrium will increase, leading to higher contractility in the atrial myocytes due to a rightward shift of the pressure-volume curve. Increased left atrial pressure, as seen in diastolic dysfunction, is known to affect transmitral flow conditions and likely partially explains the altered KE time curves exhibited by the patients in this study. Also, the LV undergoes remodeling. Pathologically enlarged LV leads to an increased residual LV volume, which previously has been quantified by 4D CMR [23]. Since volume (mass) is a factor determining the KE, higher KE during diastole was thought to be seen. However, in this material no difference was seen in diastolic average KE. In contrast, systolic KE was found to be lower in patients when indexed to EDV. The fraction KE inside vs outside the diastolic vortex was found to be lower in patients with heart failure. This could be due to the larger amount of residual LV volume, since this leads to a larger mass, not included in the vortex. In athletes with significantly larger LV compared to sedentary controls (250 ± 32 ml vs 198 ± 38 ml), there was no difference in fraction of KE inside the diastolic vortex (70 ± 1 % vs 73 ± 2 %) [21]. Thus, the LV size alone does not account for the lower fraction seen in patients in the current study. Vortex volume is inevitably coupled to stroke volume (what flows out must flow in), and the vortex has been suggested to function as an energy sink, delaying the dissipation of fluid KE into pressure [24]. KE outside the vortex may then add to the disease progression. Furthermore, in patients, 19 % of the change in KE inside the diastolic vortex can be explained by change in vortex volume, whereas in controls, 57 % of the ΔKE can be explained by change in vortex volume. Thus, in patients, the KE change is to 81 % explained by change in velocity squared, but in controls, only about half the KE change is explained by velocity squared. These relationships are true even during diastasis, when the velocities are lower.

The majority of patients in the present cohort showed left-bundle branch block (LBBB) on ECG and were under consideration for cardiac resynchronization therapy, and the dyssynchrony was expected to affect KE. Interestingly, regional wall motion in terms of presence or absence of septal dyskinesia had no impact on KE in this study.

Altered energy levels during systole was interpreted as due to abnormal flow conditions, and may contribute to increased remodeling of the heart through convective deceleration and associated pressure increase, thereby contributing to the downward spiral into advanced stages of heart failure. Abnormal flow conditions in heart failure was also discussed by Zajac et al*.* [25] who found that patients with DCM have higher turbulent KE compared to controls, however, the non-turbulent KE was not evaluated in that paper.

### Clinical implications

The KE time curve patterns in heart failure were unrelated to NYHA classification. These results highlights the issue of heart failure as a clinical diagnosis, since the degree of cardiac function impairment is poorly reflected in terms of subjectively assessed physical performance. This study shows that the mean KE in IHD can be partially explained by heart size expressed as EDV, but not by the amount of scarred LV myocardium. Thus, larger LV volume gives higher KE. This is exemplified in the patient exhibiting the highest mean KE in our study (9.1 mJ); this patient has an extremely large LV EDV of almost 750 ml.

There was only a moderate strength of agreement between KE time curve pattern and degree of diastolic dysfunction by CMR (Cohen’s kappa = 0.41) and no clear correlation between KE time curve pattern and NYHA classification or 6MWT. This indicates that KE time curves differ from established measures of cardiac function and as such constitute a conceptually new approach to the assessment of heart failure through the study of intracardiac flow.

All patients in this study were included during stable, compensated disease. Thus, it is not known whether the same findings could be seen in acutely decompensated states of heart failure or in isolated diastolic heart failure. Much is yet to be elucidated about the transition from normal myocardial function to heart failure, as well as for the transition from isolated diastolic heart failure to combined diastolic and systolic heart failure.

### Limitations

This study lacks invasive pressure measurements and therefore the cardiac load conditions are unknown. Echocardiography scanning ranged between 1 and 184 days before or after CMR (in one patient >6 years after CMR). Therefore comparison between the modalities would be misleading, and was therefore not performed. This study was also not designed to compare the two modalities. The patients included in this study were older than the controls and since metabolism has been shown to decrease with age [26], this could partially explain the lower CI in patients. The lower CI is also in line with previous studies on patients with heart failure [27]. Also, EDV, ESV, and SV shrink with age [28]. The difference in SV between patients (with lower average SV) and controls (with higher average SV) might therefore be misrepresenting to some degree, producing for example too strong differences when indexing average KE for SV. However, in order to compare different patient groups, SV is a better alternative for indexing compared to EDV (which is known beforehand to be increased in dilated hearts) and CI (which differs very little between different patient groups). This exploratory study consists of a relatively small number of patients. All patients, however, exhibited altered KE time curves compared to controls, which demonstrates the potential value of the presented technique. However, it is unknown if KE time curves are as altered in less severe heart failure or in heart failure with preserved ejection fraction, and therefore further investigation is motivated. The delineated inflowing vortex includes the inflow jet, and this could therefore have an impact on the fraction of KE inside the vortex. However, if the inflow jet would be excluded, even less KE would be found within the vortex boundary.

## Conclusions

This study shows that patients with heart failure can be divided into three kinetic energy (KE) time curve patterns, all qualitatively altered compared to controls. The KE time curves do not correlate with NYHA classification or 6-minute walk test, and may therefore constitute a conceptually new method to quantify heart failure.

## Abbreviations

2-, 3, 4ch: Two-, three-, and four-chamber view of the left ventricle
2D-flow: Two-dimensional blood flow
3D: Three-dimensional
4D-flow: Four-dimensional blood flow
6MWT: 6-minute walk test
BSA: Body surface area
bSSFP: Balanced steady-state free precession
CI: Cardiac index
CMR: Cardiovascular magnetic resonance
CO: Cardiac output
DCM: Dilated cardiomyopathy
ECG: Electrocardiography
EDV: End-diastolic volume
ESV: End-systolic volume
GFR: Glomerular filtration rate
GPU: Graphical Processing Unit
GRE: Gradient echo
IHD: Ischemic heart disease
IR: Inversion recovery
KE: Kinetic energy, ½mv^2^ where m = mass, v = velocity
LCS: Lagrangian coherent structures
LV: Left ventricle, left ventricular
LVM: Left ventricular mass
MDT: Mitral deceleration time
NYHA: New York Heart Association
PER: Peak emptying rate
PFR: Peak filling rate
SD: Standard deviation
SEM: Standard error of the mean
SV: Stroke volume
TE: Echo time
TFE: Turbo-field echo
TR: Repetition time
VENC: Velocity encoding
